# Designing a Syndromic Bovine Mortality Surveillance System: Lessons Learned From the 1-Year Test of the French OMAR Alert Tool

**DOI:** 10.3389/fvets.2019.00453

**Published:** 2020-01-09

**Authors:** Carole Sala, Jean-Luc Vinard, Fanny Pandolfi, Yves Lambert, Didier Calavas, Céline Dupuy, Emmanuel Garin, Anne Touratier

**Affiliations:** ^1^Epidemiology and Support to Surveillance Unit, University of Lyon-ANSES Lyon, French Agency for Food, Environmental and Occupational Health & Safety (ANSES), Lyon, France; ^2^National Technical Grouping of Vets Association (SNGTV), Paris, France; ^3^Ministry of Agriculture, Directorate General for Food (DGAL), Paris, France; ^4^National Federation of Farmers' Animal Health Services (GDS France), Paris, France

**Keywords:** syndromic surveillance, cattle, mortality, OMAR project, France, field test

## Abstract

Between May 2018 and 2019, a syndromic bovine mortality surveillance system (OMAR) was tested in 10 volunteer French *départements* (French intermediate-level administrative unit) to assess its performance in real conditions, as well as the human and financial resources needed to ensure normal functioning. The system is based on the automated weekly analysis of the number of cattle deaths reported by renderers in the Fallen Stock Data Interchange Database established in January 2011. In our system, every Thursday, the number of deaths is grouped by ISO week and small surveillance areas and then analyzed using traditional time-series analysis steps (cleaning, prediction, signal detection). For each of the five detection algorithms implemented (i.e., the exponentially weighted moving average chart, cumulative sum chart, Shewhart chart, Holt-Winters, and historical limits algorithms), seven detection limits are applied, giving a signal score from 1 (low excess mortality) to 7 (high excess mortality). The severity of excess mortality (alarm) is then classified into four categories, from very low to very high, by combining the signal scores, the relative excess mortality, and the persistence of the signal(s) over the previous 4 weeks. Detailed and interactive weekly reports and a short online questionnaire help pilot *départements* and the OMAR central coordination cell assess the performance of the system. During the 1-year test, the system showed highly variable sensitivity among *départements*. This variability was partly due not only to the demographic distribution of cattle (very few signals in low-density areas) but also to the renderer's delay in reporting to the Fallen Stock Data Interchange Database (on average, only 40% of the number of real deaths had been transmitted within week, with huge variations among *départements*). As a result, in the pilot *départements*, very few alarms required on-farm investigation and excess mortality often involved a small number of farms already known to have health or welfare problems. Despite its perfectibility, the system nevertheless proved useful in the daily work of animal health professionals for collective and individual surveillance. The test is still ongoing for a second year in nine *départements* to evaluate the effectiveness of the improvements agreed upon at the final meeting.

## Introduction

In France, as in many countries, animal disease control is based on a combination of active (planned) and passive (event-driven) surveillance programs and control measures, in order to achieve freedom or controlled status for the main regulatory diseases. In a context of very low prevalence or absence of disease, these traditional surveillance measures are reaching their limits in terms of effectiveness and cost/benefit ratio. In addition, in view of the increased risk of emergence, the effectiveness of traditional surveillance programs in detecting emerging diseases or the introduction of exotic diseases is questionable, as they have not been designed for this purpose. On the other hand, the evolution of agricultural practices in recent decades with the systematic recording of livestock data and the development of powerful tools for the management and analysis of large databases allows for the cost-effective use of regularly recorded information for livestock monitoring. This context has been favorable to the development of syndromic surveillance (SyS) in animal health (1).

SyS is usually defined as the real-time (or near real-time) and automated collection, analysis, interpretation, and dissemination of health-related indicators, to reveal early changes in the health status of a population or to identify the impact (or absence of impact) of potential human or veterinary public health threats that require effective public health action (2, 3). This non-specific, data-driven surveillance system is developed as a more cost-effective and earlier warning system than traditional systems (passive and active surveillance systems), but can also complement them (4–6).

Nevertheless, although SyS data can be a valuable tool for public and animal health practices, few systems are currently operational in animal health (7). Despite the substantial number of methodological developments involving SyS, there are few fully operational SyS systems and few field feedback experiences about SyS systems especially in animal health (8–11). This contradiction reveals the difficulties in (i) convincing public or private entities to fund the evaluation of these systems in the field, (ii) finding the business model for operational deployment, and (iii) designing these systems in such a way that they are not limited to large health services that have sufficient expertise and staff to review alarms. Evaluations of human SyS systems have shown that the usefulness of the results depends highly on the expertise of human resources locally (10, 12, 13). When designing SyS systems, little attention is generally paid to how easily local health professionals can exploit the results, particularly because they may have different levels of expertise. The ease of result interpretation is a limiting factor in the value of SyS systems, because national or regional animal health services are often too far removed from the local level for satisfactory use of the results.

In France, we are developing a SyS system for bovine mortality, called OMAR (*observatoire de la mortalité des animaux de rente*), to identify significant excesses of deaths in the bovine population potentially linked to emerging diseases, epidemiological changes in the pattern of enzootic diseases, or other health events. We designed a pilot application of the system within a collaborative, multi-stakeholder working group of the French platform for epidemiological surveillance in animal health (ESA Platform, https://www.plateforme-esa.fr/). We paid particular attention to its use in the field, adapting the frequency and format of reports to the organization of animal health management units, their expectations, human resources, and expertise.

Since 2018, we have implemented a 1-year pilot phase to (i) test, in real-life situations, the system's design; (ii) assess its global performance (sensitivity, specificity, precocity, utility); (iii) evaluate the human and financial resources required; and (iv) identify any changes needed in terms of regulatory support to ensure the normal functioning of the system. The purpose of this article is to describe the methodology and design of our SyS system and to present the results of the 1-year test performed in real conditions. We also discuss the advantages and limitations of the system from the point of view of local and national animal health services.

## Materials and Methods

We use Toad for MySql 7.5.0.966, MySQL, R 3.3.3, and RStudio 1.1.383, to manage and analyze the data, and Perl 5.26.1 and the Windows tasks planner for automated task.

### Organization of the 1-Year Test Period

**Figure 1:**
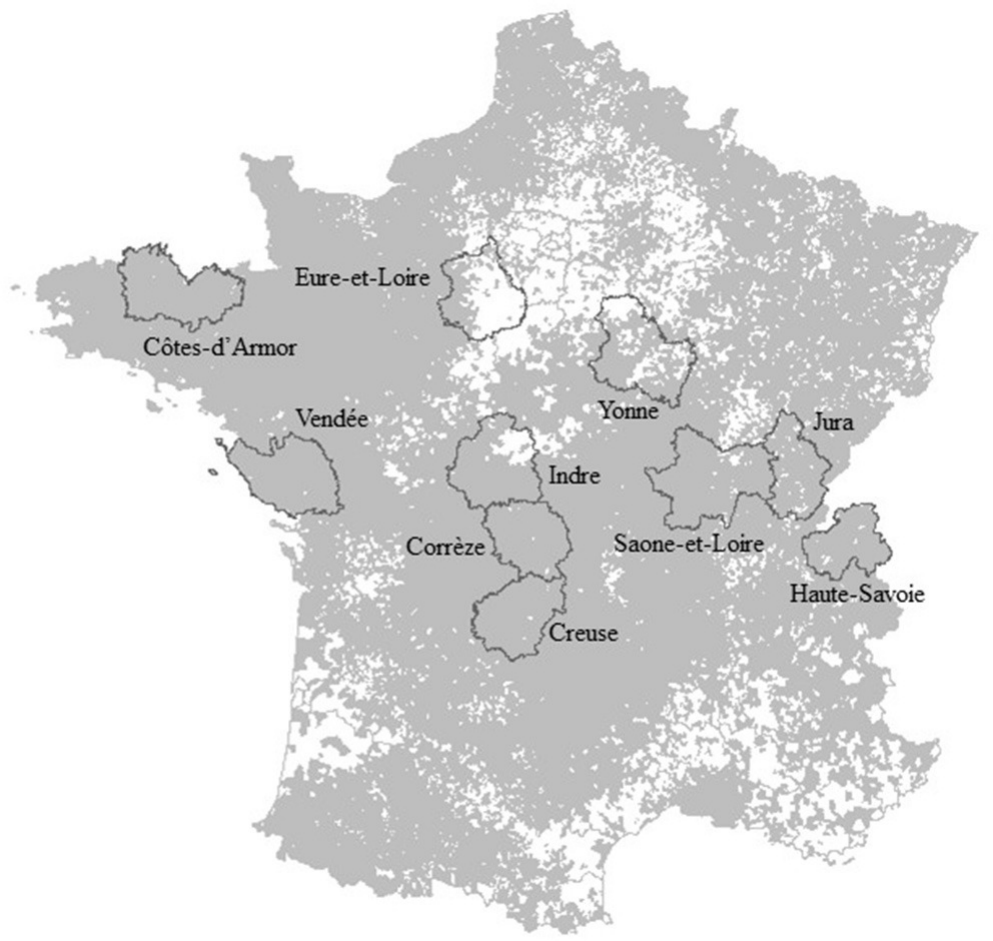
Code number and names of the 10 pilot *départements* (dark gray line) and surveillance areas (gray) analyzed during the 1-year test period.

**Figure 2:**
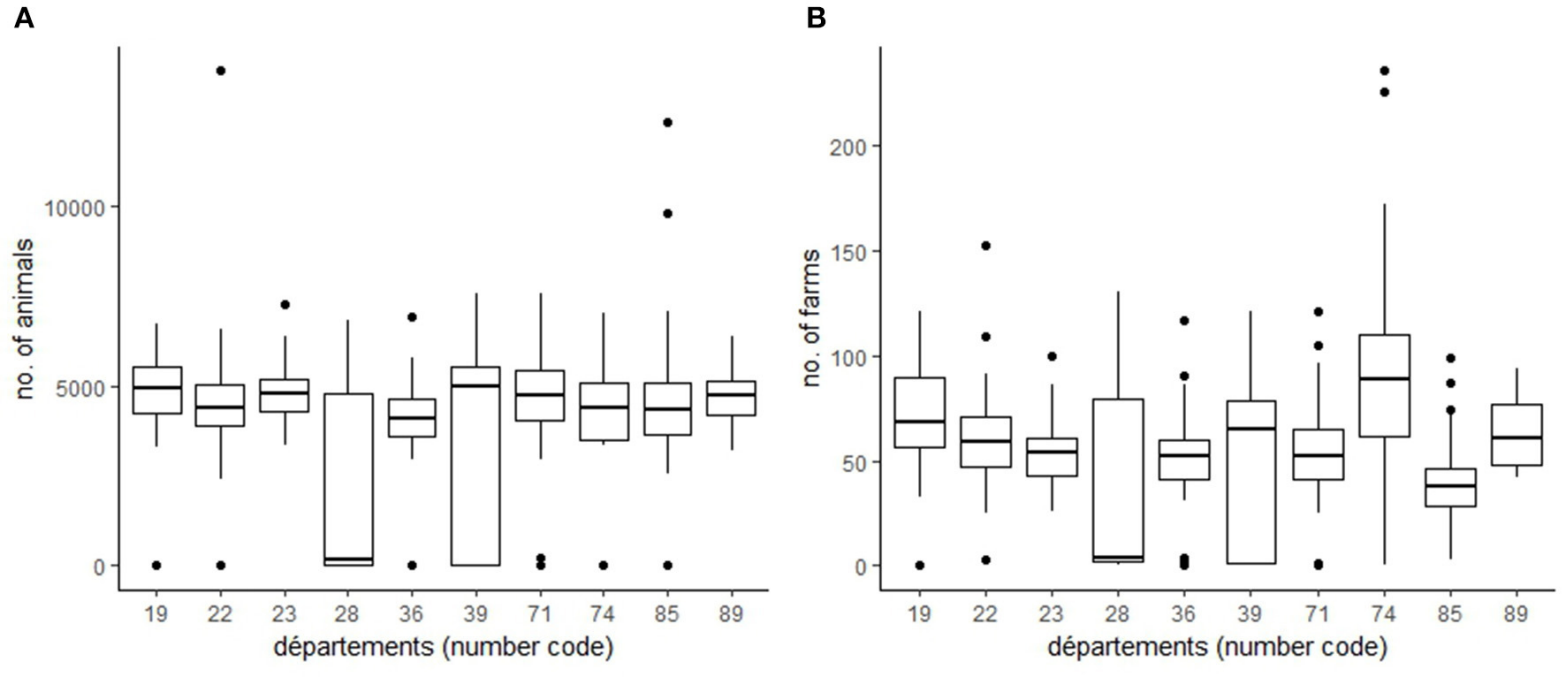
Distribution (boxplot) of the annual number of animals **(A)** and farms **(B)** of surveillance areas in each pilot *département*.

A national call was launched in January 2018 to select a limited number of volunteer *départements*. These pilot *départements* had to demonstrate the mutual commitment of the three main local animal health services [the departmental animal health office (*service vétérinaire départemental*, or DDecPP), departmental farmers' animal health service (*Groupement de défense sanitaire*, or GDS), and departmental technical grouping of vets association (*Groupement Technique Vétérinaire*, or GTV)] to facilitate field investigations, if needed. In addition, regional voluntary services could participate in support of departmental services. Ten departments expressed interest in participating and met the criteria: *Corrèze* (number code 19), *Côtes-d'Amor* (22), *Creuse* (23), *Eure-et-Loire* (28), *Indre* (36), *Jura* (39), *Saone-et-Loire* (71), *Haute-Savoie* (74), *Vendée* (85), and *Yonne* (89) (Figure 1). Three of them were involved in the Omar project since its inception (*Corrèze*, Côtes-d'Amor, and *Yonne*) (14).These *département*s represented the various cattle breeding contexts in France: (i) low, intermediate, and high cattle density areas (Figure 2); (ii) traditional and intensive breeding practices (including mountain pasturing); and (iii) dairy and beef production types (15).

**Figure 3:**
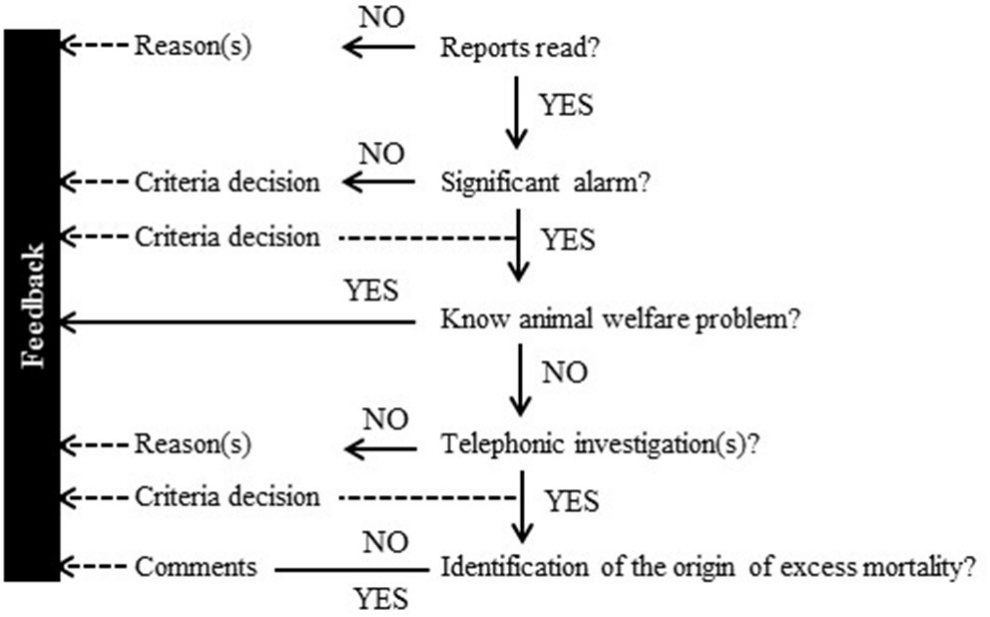
General scheme for report interpretation and feedback.

The national OMAR coordination cell (OMAR-NC) organized a 1-day meeting in April 2018 to present the system, the objectives, and expectations of the test phase and to train the health professionals (report reading method, extraction of main information, etc.). About 40 people from the three local animal health services in each volunteer department and associated regional levels attended the meeting. The coordination and organization of the various services within each *département* was unregimented, but a leader was designated in each *département* as the main departmental coordinator and in charge of reporting to the OMAR-NC. A general scheme for report interpretation (Figure 3) and a short online questionnaire (Supplementary Material S1) helped standardize the weekly report. A mid-term meeting was held in November 2018 to present and discuss local organizations (who, how, time spent, etc.), uses of the results (how, for what, etc.), and limitations in each *départements*. This meeting was also used as an opportunity to adapt and improve the system during the period of test, if necessary. A final meeting was held in June 2019, to discuss the statistical results, the feedback, and improvements needed and to conclude on the interest (or not) in continuing to test the system.

### Data Sources

The Fallen Stock Data Interchange (FSDI) database forms the basis of the system. This database centralizes the information of the removal visits transmitted daily by each of the 56 renderer collection points in mainland France. The centralization has been all-inclusive since January 2011. We used information on

- the identification number of the holding for which the removal is requested and its location at the *commune* scale (smallest French administrative unit, hereafter referred to as “municipality”);
- the number of cadavers, their identification number(s), age group, weight, and location (municipality); and
- the date of the removal request (considered as the date of death) and date of removal.

This information is of high quality because it is used for payment of rendering fees by the French dead-on-farm animal service.

Our SyS also uses the National Cattle Register (NCR) database because it contains all the information about the holdings (identification number, location, species kept) and animals (sex, breed, movement). It also provides demographic information (number of holdings, average number of females 2 years and older, number of cattle slaughtered, and births) for each farm and municipality (15). It also serves to cross-check the FSDI database because the two databases are not interconnected, and data such as farm identification numbers are sometimes not updated in the FSDI database.

### Spatial and Temporal Units

To manage the heterogeneity of cattle density and because the precise geolocation of farms is not available, we merged the 35,756 French municipalities into 11,377 larger spatial units. This pooling scheme ensures a sufficient number of animals and deaths in each area to limit statistically random variation due to small sample sizes. It also homogenizes, as much as possible, the number of animals between spatial units to obtain an almost identical alarm probability for each spatial unit and for an equivalent health event. Our algorithm merges contiguous municipalities with cattle until each new spatial unit reaches an annual average population of at least 3,000 animals (equal to the average number of females 2 years and older and number of cattle slaughtered, calculated from the NCR) and/or there are no more municipalities or units to merge. The algorithm primarily merges municipalities within the same *département* but does not limit the merging to within *département*. Some of the spatial units thus occupy several departments. The algorithm uses the geographical file from the French National Institute of Geographic and Forest Information and demographic information from the NCR. It iteratively constructs a new file with a new data table of demographic information and list of the municipalities in each new spatial unit. Detailed aggregation algorithm rules are available in Supplementary Material S2.

Although the data are available daily, our SyS system operates on a weekly basis to ensure a sufficient number of deaths in each surveillance area and to be consistent with the human resources that can be assigned to the system at departmental and national levels. We aggregate the number of deaths by ISO week, from Monday to Sunday, using the date of the removal request.

### Analysis

The OMAR system is designed to identify sudden increases in mortality (a one-time significant difference between observed and predicted deaths) as well as slow deviation, i.e., systematic deviation from the expected value, which is not significant over a week but significant over several consecutive weeks. To do so, we use five detection algorithms: the Holt-Winters algorithm (16), “historical limits” algorithm from the Center for Disease Control (17), and Shewhart algorithm for sudden increases and the exponentially weighted moving average (EWMA) and cumulative sum (CUSUM) algorithms for slow deviations (18).

Every Thursday of week *w*_+1_, our OMAR tool evaluates the mortality in the immediately previous week *w*. The analysis is scheduled for 00:30 am, and the results are generally available around 8:00 am. The analysis follows classical steps for SyS systems: (i) preparing the data and time series (TSs), (ii) cleaning the TSs from historical anomalies, (iii) predicting the expected values, and (iv) comparing expected and observed values to detect potential excesses.

The particularity of our system is that we record all the elements (timetable, data, and results of the analysis), in an S4 object oriented system, adapted from R codes and the approach used in the Vetsyn package developed by Dorea et al. (19). Details of our S4 object are provided in Supplementary Material S3.

#### Step 1: Data Processing

Data on cattle removals from January 2011 to week *w* are extracted from the FSDI database and prepared for the analysis according to the following steps:

♦ Exclusion of attempted removals (cadaver not found, i.e., number of removal animals and weight equal to 0)
♦ Cross-checking of holding identification with the NCR database and identification of holdings that are not farms (veterinary clinics and schools, laboratories, slaughterhouses, etc.) to distinguish them
♦ Re-coding of age groups into four categories (under 21 days, 21 days–under 6 months, 6 months–under 24 months, 24 months and over)
♦ Correction of the removal municipality identifier to ensure perfect consistency with geographical files due to regular administrative changes in French municipalities
♦ Coding municipalities into new surveillance area
♦ Coding the date of the removal request into its ISO week

The number of deaths is then aggregated by ISO week and surveillance area as defined in the *Spatial and Temporal Units* section. There were as many TS as there were surveillance areas each week.

To gain calculation time, only TSs having at least one death recorded in the FSDI over the last 4 weeks (w_−3_, w_−2_, w_−1_, w) are kept for analysis.

From this point, each TS_w_ is decomposed into four sub-times series (STS): STS_w−3_ = t_0_ to t_w−3_, STS_w−2_ = t_0_ to t_w−2_, STS_w−1_ = t_0_ to t_w−1_, and STS_w_ = t_0_ to t_w_, with t_0_ = 2011-W01 for the historical limit and t_0_ = w – (5 ^*^ 52) for the other algorithms.

#### Step 2: Creation of Cleaned Baselines

To clean the STS, the observed values are compared to the value predicted by a general linear model (GLM) and those above the 95% CI are lowered to this value.

For each STS, the GLM is fitted on the entire TS (i.e., from 2011-W01). Formulae tested includes *a minima* a sinusoidal annual seasonality. Linear trend and autoregressive components over the last 4 weeks are also tested. The selection of the best formula is based on the Akaike information criterion or quasi-Akaike information criterion depending on the GLM family selected. To determine the GLM family, the model is first fitted to a Poisson family and the best formula is tested for the over dispersion. In case of over dispersion, a generalized Poisson family is tested; if there is a convergence problem, a quasi-Poisson GLM is tried, and if there is still a convergence problem, a negative binomial GLM is used.

#### Step 3: Calculation of the Expected Mortality for Week x (x = w_−3_…w)

Three methods are applied to calculate the expected mortality, depending on the detection algorithm:

◦ For the control charts (EWMA, CUSUM, and Shewhart algorithms), the expected mortality is estimated from the predicted value of the GLM using the family and formula selected in the cleaning step (Step 2). The model is rerun on the cleaned baseline of the last 5 years. A 4-week guard is applied to limit contamination of the past by potential recent health events.
◦ For the Holt-Winters algorithm, a 5-year baseline with a 4-week guard is also used. The values of the smoothing parameters for weight (α), trend (β), and seasonality (γ) are determined first testing the optimizer implemented in the Holt-Winters function of the stats R package (20); in case of failure, β is set to 0.1 or removed if no trend is detected by the GLM, and α and γ are estimated by the optimizer; in case of failure again, α, β, and γ are set to 0.1.
◦ For the historical limits algorithm, the expected value for the last 4 weeks is calculated from the average values observed over 12-week periods (three blocks of 4 weeks) centered on the index of the week of interest (for index week x, the period is x – 7 to x + 4) over the last Y complete years (Y ≥ 5) (21).

#### Step 4: Detection of Excess Mortality in Week x (x = w_−3_…w) and Signal Scoring

For each STS and algorithm, the observed value in week x is compared to the upper confidence limit predicted by the algorithm at the threshold T. Seven upper confidence limits are used to obtain seven thresholds T and score the signal from low (1) to high (7) excess mortality.

For the Holt-Winters and historical limits algorithms, the observed value is compared with the upper confidence limit predicted by the algorithm at the threshold T. Seven values of SD are used from 1.65 to 3.25 to obtain seven thresholds T and score the signal from low to high excess mortality.

The control charts are applied to the residuals of the GLM due to the seasonality of mortality on French farms linked to birthing seasons and farming practices. The Shewhart method is parameterized with the default value of the qcc R package with k (the number of SDs allowed above the average), set to 2 and a classical calculation of the SD. The EWMA algorithm is parameterized to detect low increases in mortality with the constant adjustment λ set to 0.2 to give more weight to the oldest values. For the CUSUM method (h the amount of shift to detect in the process) measured in SEs is set to 1. For the three control charts, seven values of SD from 2.33 to 3.75 are used to obtain seven thresholds.

The system gradually compares the observed mortality value with each threshold of each algorithm and increase its score by +1 at each exceeded threshold. At the end of the process, each algorithm has a signal scored from 0 (no statistical excess mortality = no signal) to 7 (the observed value exceeds the seventh upper limit).

#### Steps 2′, 3′, and 4′ for TS With a Median Number of Deaths Under One

The system makes use of the location of death, which is not always the location of the holding, because some of the surveillance areas do not have a (permanent) bovine population. In some of these areas, mortality cases are rare, but monitoring them may be of great interest to detect, for example, seasonal health events that cause grouped cases of mortality, such as anthrax in mountain pastures. We use a very basic system to detect excess mortality for STSs with a majority of zeros (median under one): no cleaning or any detection algorithm is applied, but the observed data are compared to set values ranging from 3 to 9 to score the signal from 1 to 7.

#### Step 5: Alarm Classification Severity

To limit the sensitivity of the analysis, increase its specificity, and help identify excess mortality (alarm) that requires follow-up or investigation, the OMAR system classifies the severity of the excess mortality according to

▪ the number of algorithms with a score above 0: high consistency between the results of the different algorithms indicates high likelihood of the alarm (i.e., the excess mortality);
▪ the signal scores for week *w* for each algorithm and type of algorithm: increases in the global scores indicate increases in excess mortality;
▪ the change in signal scores over the last 4 weeks to identify excess mortality in the process of worsening; and
▪ the level of excess mortality and its change over time to identify worsening excesses and/or very significant mortality excesses.

We thus evaluated 25 non-exclusive criteria based on a 2-year retrospective analysis of real data in five representative *départements* (Table 1). We classified them into four categories, considering that the less frequent the criterion, the more serious the alarm. We obtained 3 low severity criteria, 6 medium severity criteria, and 10 high severity criteria (Table 1). Three criteria were not included because they were too rare. All other results were considered very low severity. For each area with an alarm, when different severity criteria were present, we reported the severity of the alarm corresponding to the highest severity criterion.

**Table 1:**
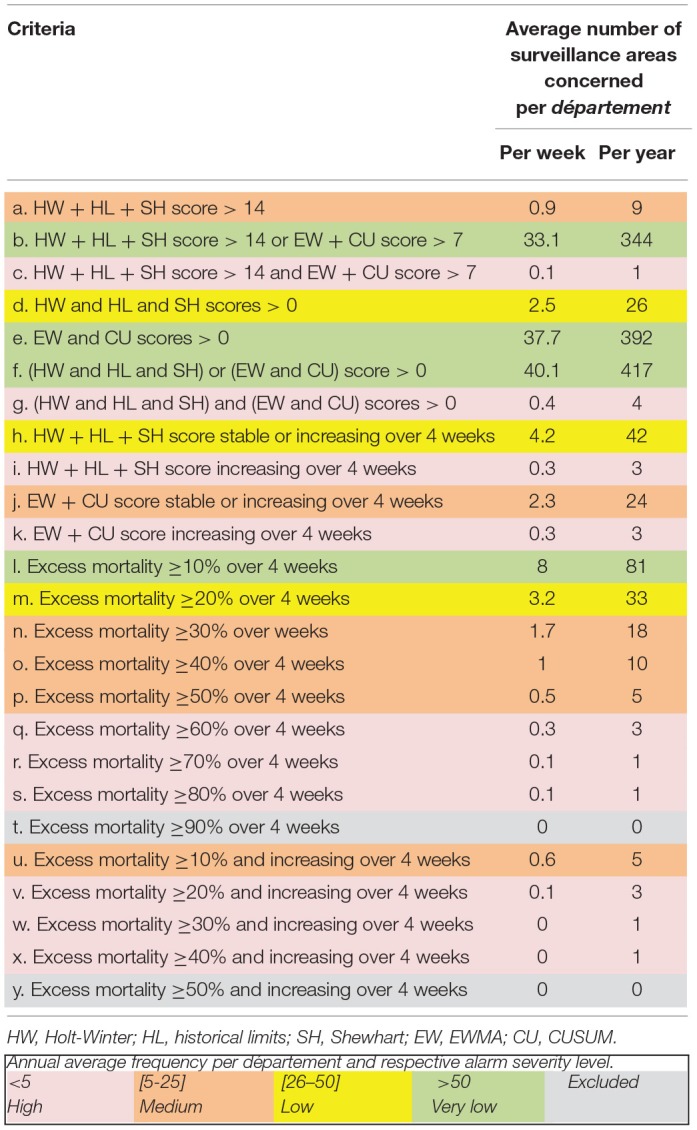
Criteria used to classify the severity of excess mortality: results of a 2-year (2016–2018) retrospective analysis in 277 surveillance areas from five French *départements* (Côtes-d'Armor, Corrèze, Puy-de-Dome, Vosges, and Yonne).

### Reports

Each week, the system produces one national and one departmental report for each pilot *département*, regardless of the results of week *w* (with or without alarms). Reports are automatically uploaded to the secure https site managed by ANSES. In parallel, an email is automatically sent to a predefined list of recipients in each *département*, indicating the availability of the reports and summarizing the results (number of areas with an alarm and number of areas per alarm severity).

#### Départemental Report

The departmental report gives the results for the surveillance areas within the *département*, and for any overlapping areas (see the “*Spatial and Temporal Units*” section). It contains information not only for assessing the severity and degree of the excesses mortality, but also for their field investigation. Detailed confidential information on cattle mortality in farms in alarm areas is provided, but access to this information is limited to authorized health services (DDPP and GDS).

The departmental report consists of three files with different levels of confidentiality.

a) Summary file

**Figure 4:**
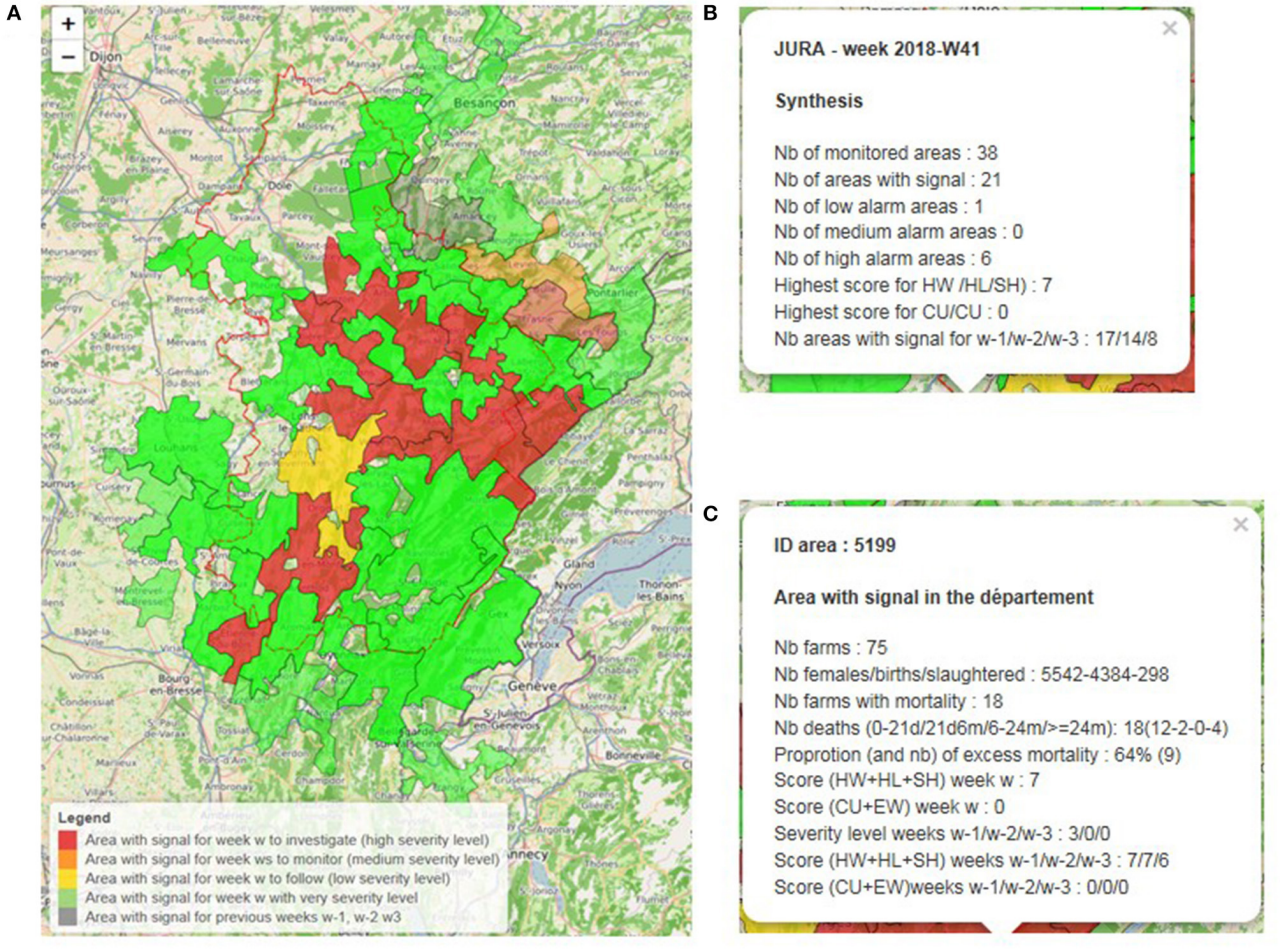
Example of a departmental summary report: results for Jura (39) for week 2018-W41 showing global map **(A)**, departmental display box **(B)**, and area display box **(C)**.

This is an interactive (leaflet) map providing a visual report on mortality in the *département* and in adjacent surveillance areas (Figure 4A). It shows areas color-coded by alarm severity (green = very low, yellow = low, orange = medium, red = high) and also provides summary information in display boxes that appear when the map is clicked on the following:

◦ The departmental display box gives the total number of areas in the *départment*; number of areas with an alarm during the weeks *w, w_−1_, w_−2_*, and *w_−3_*; number of areas per alarm severity for week *w*; and highest score by algorithm type (fast/slow) for week *w* (Figure 4B).
◦ For areas within the *départment* with an alarm during week w, the display box provides the area identifier (ID), number of cattle farms, number of cattle farms with mortality during week *w*, number of cattle deaths during week *w* (total and per age groups), and score per algorithm type for weeks *w, w_−1_, w_−2_*, and *w_−3_* (Figure 4C).
◦ For areas within the *département* that had an alarm during the previous 3 weeks and no longer had a signal during week *w*, the display box indicates the score per algorithm type for weeks *w*_−1_, *w_−2_*, and *w_−3_*. These areas are shaded in gray on the map (Figure 4A).

Because this file does not contain confidential information, it is accessible to all animal health stakeholders involved in the testing phase in each pilot *département*.

b) Detailed file

Containing confidential information, the access to this Excel file is restricted to authorized animal health services in each pilot *département*. It gives detailed information on cattle mortality per area and farm during the last 4 weeks, in six sheets (Supplementary Material S4):

◦ The “Alarm w” sheet gives information on areas with an alarm for week w. We detail the alarm identification number (ISO week + area identification number); area identification number; number of animals and farms; number of farms with mortality; number of deaths (total and per age group); criteria that led to the alarm classification severity for week *w*; number of algorithms with a score over 0 for each weeks *w, w*_−1_, *w_−2_*, and *w_−3_*; percentage and minimum and maximum number of the excess mortality; and score for each detection algorithm for weeks *w, w_−1_, w_−2_*, and *w*_−*3*_.
◦ The “Alarm −1 to −3” sheet provides information on the area for which an alarm was detected over the previous 3 weeks *w*_−1_*, w_−2_*, or *w_−3_*during the reanalysis in week *w*; information is the same as that of “Alarm w” sheet, except for the severity criteria which are not calculated.
◦ The “Farms w” sheet provides information on farms with mortality in area with an alarm during week *w* to help identify the source of the excess mortality. For each farm having requested a removal, we detail the alarm and area identities; the identification number, name, municipality, and group type (15) of the farm as recorded in the NCR database; the identification number, name, and municipality of the farm as recorded in the FSDI database; the municipality of removal; the standardized mortality ratio calculated for the last 1-year period (from the NCR database); average number of animals in the last 1-year period (from the NCR database); number of removals during week *w* (total and by age group); number of removals during the last 4 weeks and for the same 4-week period but the previous year; and identification number of the health veterinary (veterinary in charge of the control of regulatory diseases in the herd).
◦ The “Farms −1 to −3” sheet provides the same information as the “Farms w” sheet, but for farms in areas with an alarm during the previous 3 weeks *w*_−1_*, w_−2_*, and *w_−3_*upon reanalysis. The mortality per 4-week blocks is not provided.
◦ The “Area info” and “Municipality info” sheets give information as well as links between areas (spatial units obtained from the aggregation algorithm) and municipalities (real administrative unit).
c) Follow up file

**Figure 5:**
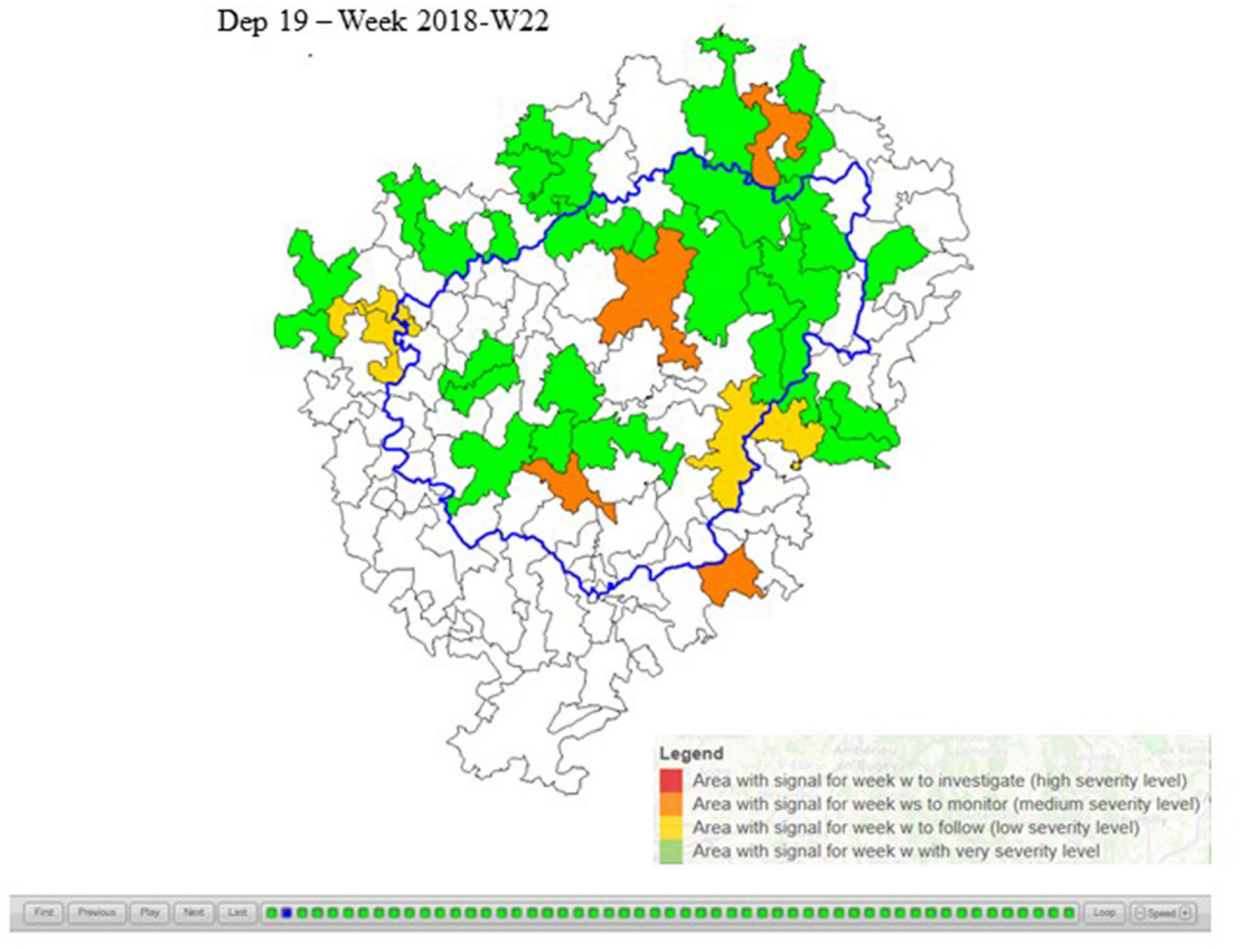
Departmental follow-up report for Corrèze: image for week 2018-W22.

This interactive html map (Figure 5) facilitates the follow-up of the alarm area over time. It is updated each week with the result of week *w* and shows week by week the alarm areas per severity. A toolbar is available to scroll manually or automatically through each week. It helps animal health services to monitor the spatio-temporal dynamics of excess mortality and to identify worsening patterns (increase in severity over time, progressive grouping of alarm areas, and excess mortality spatial shifts through the *département*, etc.). It is accessible to all animal health stakeholders involved in the testing phase of each *département*.

#### National Report

**Figure 6:**
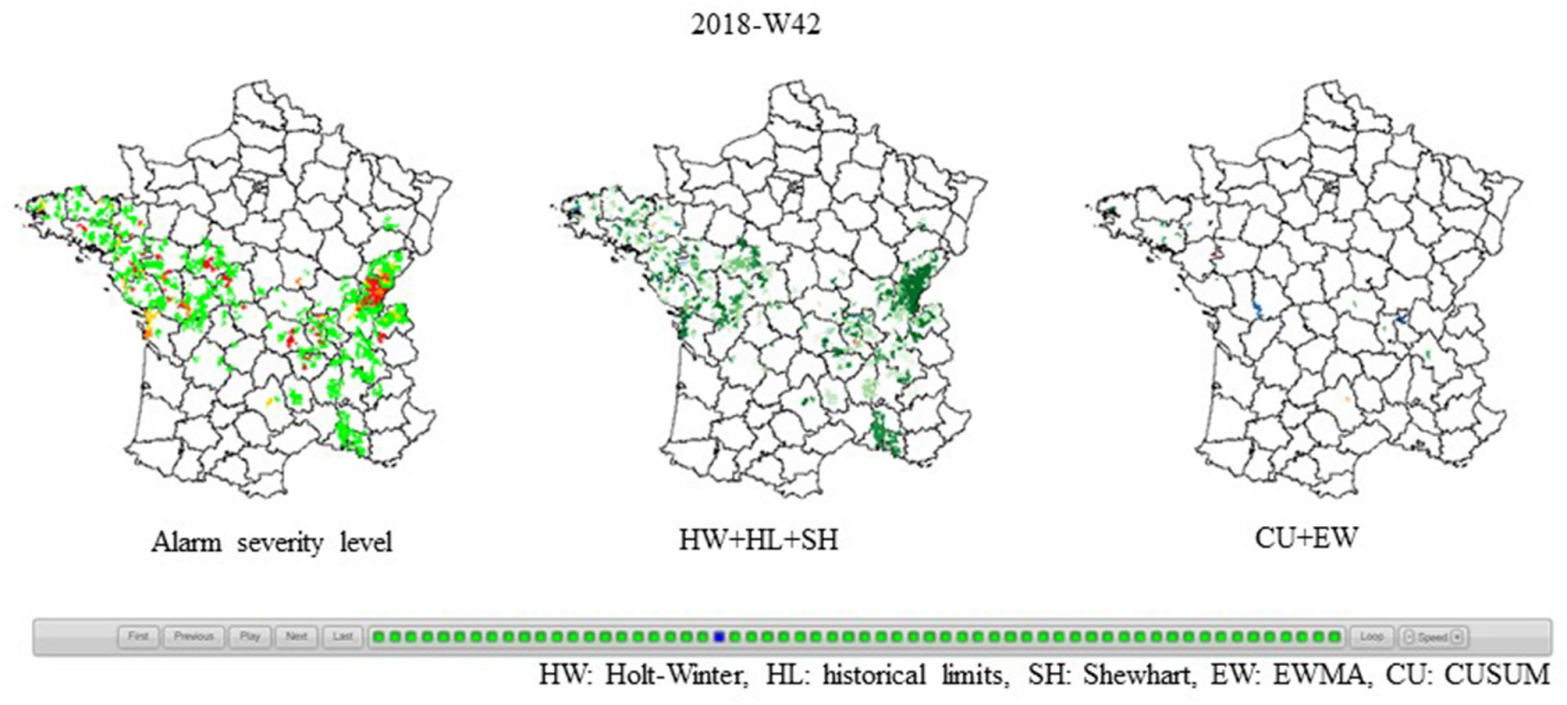
Example of the national report: results for ISO week 2018-W42, per alarm severity (**Left**), global score [from 1 (light green) to 21 (dark green)] for algorithms detecting sudden increases (**Middle**), and global score [from 1 (light green) to 14 (dark green)] for algorithms detecting slow increases (**Right**).

The national report is similar to the follow-up report of the departmental file (html map, Figure 6) but extended to the national level. It provides an overview of the spatio-temporal changes in bovine mortality week by week across all of mainland France. It is made available to the OMAR-NC and national rendering companies, so that they can have an overall quantitative view of mortality and validate their observations.

## Results and Discussion

The 1-year test period (hereafter called “test period”) began on 21 May 2018 (week 2018-W21) and ended on 19 May 2019 (week 2019-W20).

Before the beginning of the test, we checked the time it took for data to be reported in the FSDI database to assess the reality of “real-time” monitoring and determine the best day of week for analysis. Legally, the renderers have 2 full days after the request to collect the cadavers and a maximum of 7 days after the removal visit to report the information to the FSDI. Nevertheless, the analyses revealed longer delays in full data reporting with high variation among the collection centers and thus *départements* (generally, one or two different centers operate in each *département*) (delay distribution is available in Supplementary Material S5). We present and discuss the results in light of this situation.

### Results From Data and Statistical Point of View

We reanalyzed every week of the previous 4 weeks to take into account the ongoing updating and correction of data in the FSDI, as well as delays in data transmission. After discussion, we chose Thursday as the best compromise between reporting time and human resources.

Among the 11,377 surveillance areas defined by the spatial aggregation of municipalities, 8831, including 435 with cattle, had no mortality recorded over the period, and were not monitored. This high percentage of areas without animals (74%) is the due to our merging method (municipalities without animals cannot merge and stay small) and the heterogeneity of cattle density in France (Figure 1).

Over the test period, we analyzed 137,056 TS including 26,995 TS in the pilot *départements*. These TSs involved 2932 surveillance areas including 157 areas without animals or farms, and 553 areas located in pilot *départements*. Among the TSs, <5% had a median number of deaths under one. The number of TS analyzed each week was relatively constant over time, between 2,586 and 2,707, and the majority of surveillance areas showed mortality recorded every week (85%, *n* = 2,492). Similar results were observed in pilot *départements*, but the number of TSs weekly analyzed varied greatly among them due to the heterogeneity of the number of surveillance areas, linked to cattle and farm density (Figure 2) and mortality observed at the time of analysis (Table 2).

**Table 2:** Distribution of the percentage of deaths reported at the time of analysis at the national and departmental levels (*n* = number of surveillance areas).

	**France (*n* = 2932)**	***Départements number*** codes^*^ **(*****n*** **=** **553)**									
		**19**	**22**	**23**	**28**	**36**	**39**	**71**	**74**	**85**	**89**
Minimum	0	0	0	0	0	0	0	0	0	0	0
1st quartile	0	0	60	63	0	50	23	50	0	56	0
Median	31	29	80	89	0	80	67	82	73	80	0
Mean	41	38	75	80	3	73	58	71	58	76	13
3rd quartile	83	71	100	100	0	100	92	100	92	100	17
Maximum	800	400	775	300	100	200	162	229	233	600	133

* *19 Corrèze, 22 Côtes-d'Amor, 23 Creuse, 28 Eure-et-Loire, 36 Indre, 39 Jura, 71 Saone-et-Loire, 74 Haute-Savoie, 85 Vendée, 89 Yonne*.

**Figure 7:**
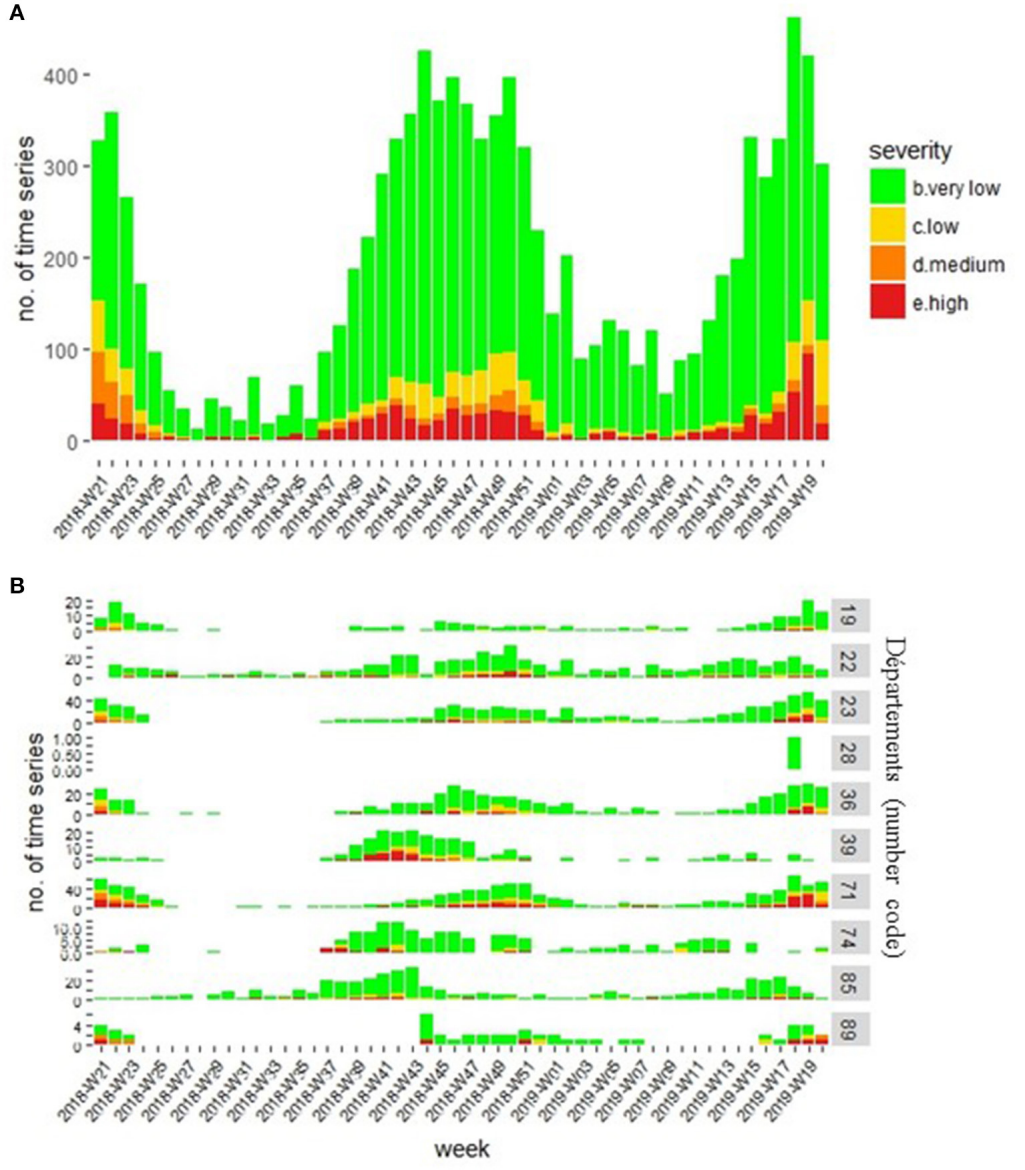
Number of time series per week and severity level at the national level **(A)** and per pilot *département* **(B)**.

Due to our inclusion criteria in the analysis process (TSs with mortality over the previous 4 weeks), 46% of TSs analyzed (*n* = 63,239) had no mortality recorded for week *w* at the time of analysis (Table 2). Among the 73,817 TSs having mortality during week *w*, no excess mortality was detected for 86% of them (*n* = 63,536). We identified very low excess mortality severity for 11% of TSs (*n* = 8,308), low severity for 1% (*n* = 766), medium severity for 0.5% (*n* = 384), and high severity for 1% (*n* = 823) (Table 3). Very low and low level severity alarms mainly originated from historical limit algorithm, known to be very (too much) sensitive (17). Nevertheless, this algorithm has proven its interest in monitoring major diffuse mortality phenomena with very low local excess mortality in France (Sala C, personal communication). Table 1 provides an overview of the global high sensitivity of the system. The number and proportion of TSs per severity level varied weekly (Figure 7). Similar results were observed in pilot *départements*, but the weekly number of areas with an alarm varied greatly among *départements* (Table 3 and Figure 7B). We detected excess mortality only once in Eure-et-Loire (28), but in Creuse (23), Saone-et-Loire (71), and Indre (36), we observed a high number of TSs with an alarm (20%).

**Table 3:** Global results over the test period at the national level and per pilot *départements*: number of times series (TS) analyzed and repartition per alarm severity (percentage among TS with mortality).

	**France**	***Départements*** number codes^**^									
TS^*^		**19**	**22**	**23**	**28**	**36**	**39**	**71**	**74**	**85**	**89**
Total	137,056	2,763	4,592	3,484	783	2,361	1,766	4,829	1,192	4,851	1,196
No mortality	63,239	1,118	127	73	737	133	397	515	352	172	813
No alarm	63,536 (86)	1,479 (90)	3,933 (88)	2,646 (78)	45 (98)	1,785 (80)	1,157 (85)	3,395 (79)	685 (82)	4,235 (91)	332 (87)
Very low severity	8,308 (11)	147 (9)	457 (10)	602 (18)	1 (2)	347 (16)	160 (12)	612 (14)	132 (16)	401 (9)	38 (10)
Low severity	766 (1)	11 (1)	32 (1)	70 (2)	0 (0)	39 (2)	19 (1)	97 (2)	8 (1)	19 (0)	3 (1)
Medium severity	384 (1)	5 (0)	7 (0)	29 (1)	0 (0)	18 (1)	6 (0)	70 (2)	2 (0)	7 (0)	4 (1)
High severity	823 (1)	3 (0)	36 (1)	64 (2)	0 (0)	39 (2)	27 (2)	140 (3)	13 (2)	17 (0)	6 (2)

* *Some time series are common to several départements*.

** *19 Corrèze, 22 Côtes-d'Amor, 23 Creuse, 28 Eure-et-Loire, 36 Indre, 39 Jura, 71 Saone-et-Loire, 74 Haute-Savoie, 85 Vendée, 89 Yonne*.

**Figure 8:**
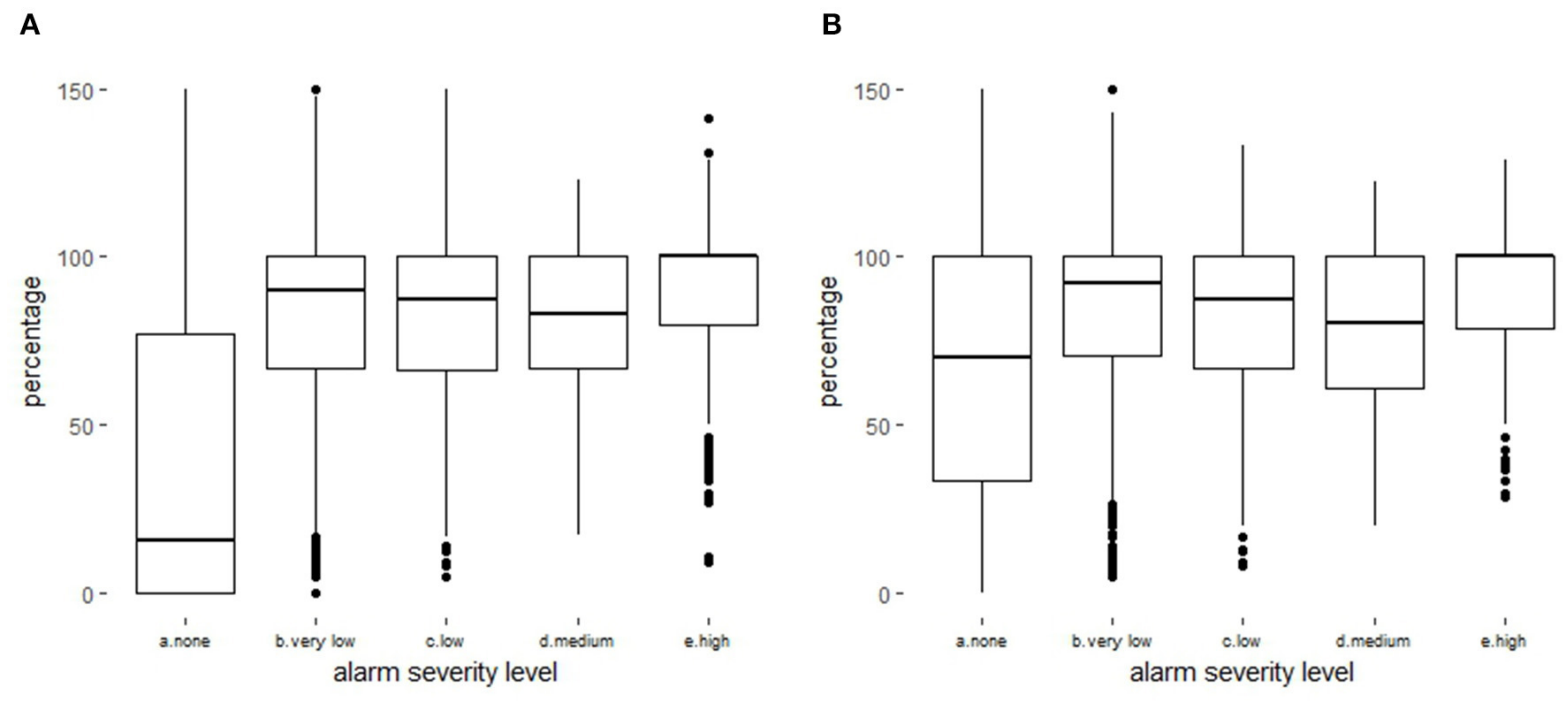
Distribution (boxplot) of the percentage of the deaths available at the time of analysis according to the severity of the alarm at national level **(A)** and in pilot *départements* **(B)**.

We first assumed that the difference in results among the pilot *départements* was due to the heterogeneity of demographics (more alarms in areas of high cattle density) and data transmission (fewer alarms in areas with longer reporting time), but this hypothesis was not strong enough to explain the low number of alarms obtained in Eure-et-Loire. When preparing the final meeting, we re-extracted the mortality from the FSDI database from the beginning of the test period to obtain the real mortality, to compare it with the mortality available at the time of the analysis (hereafter called “observed mortality”). This comparison revealed that the delay in data reporting involved not only time but also the number of deaths, leading to significant under-reporting of the mortality at the time of analysis (Table 2). Although real mortality was relatively constant over time, the amount of information available at the time of analysis varied greatly among areas and weeks (Supplementary Material S6). We thus observed very high variability between pilot *départements*, and no data had been reported at the time of analysis in Eure-et-Loire. This explains the absence of signals during the test period in this *département*. In fact, the data reporting delays had a major influence on the detection of excess mortality and alarm severity, especially for high severity (Figure 8). Nevertheless, the amount of data available in pilot *départements* was generally higher than that observed at the national level. Finally, we observed that real reporting times had a limited impact on the number of TSs analyzed. We calculated that only 729 TSs (0.5%) were not analyzed due to the absence of observed mortality.

### Results and Experience From Animal Health Services

The objectives were to draw up a general flowchart, to evaluate the effectiveness of the reports, and the advantages, drawbacks, and performance of our SyS system. An important point was the estimation of the time to consult and interpret the reports for the calculation of the financial resources required by the system. Due to insufficient human resources at the local level and the lack of financial and legal support, we limited the work of animal health professionals to consulting and interpreting reports, telephone surveys on alarms of interest (if possible), and weekly feedback via a short online questionnaire. During meetings, we also asked for feedback on the local organization, difficulties, and the usefulness of the system.

Organization at the local level was similar in the 10 pilot *départements*, with one person in charge of interpreting the report, carrying out the surveys and responding to the questionnaire. In the absence of the leader, an alternate did the work. In most *départements*, the GDS was the leader, possibly alternating with the DDecPP. Weekly results were discussed with all the other animal health stakeholders, as necessary, to identify the source of the excess mortality.

**Figure 9:**
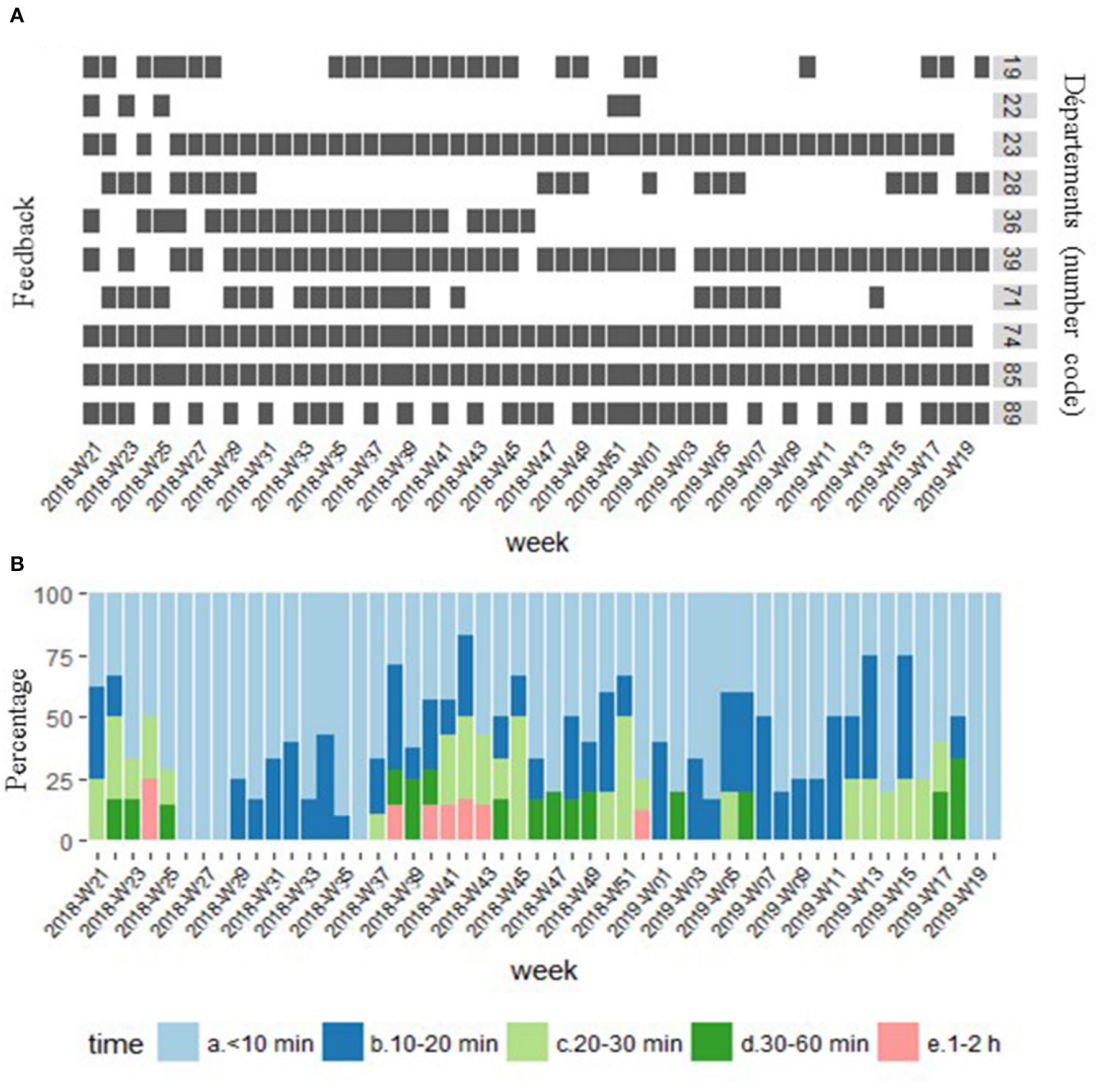
Feedback per pilot *département* and week (gray box indicates response from *département* leader) **(A)** and distribution of time dedicated to reading the report each week **(B)**.

Between 4 June 2018 and 29 May 2019, 343 of the 520 questionnaires expected were filled out, with high variability among *départements* (Figure 9A). The lack of feedback was attributed to unexpected limitations in human resources (sick leaves), high workloads with variable local human resources, and the requirement to complete the questionnaire immediately, whereas the interpretation of the results and investigations could take several days.

The 343 responses indicated that the reports were consulted in their totality (summary and detailed reports) in 87% of the cases. The two reasons for not consulting the reports were the absence of alarm areas (56%) and the low level of the severity of the alarm (44%). Lack of time was never mentioned, but the lack of time to complete the questionnaire may have biased the responses. As initially estimated, the animal health service needed <30 min to analyze the reports in 90% of cases (60% <10 min) (Figure 9B). The time to interpret the reports appeared correlated with the number of medium and high severity alarms, with an analysis time of <20 min in the absence of alarms at these levels.

Over the test period, the analysis of the reports led to 44 telephone investigations, mainly with veterinarians (*n* = 33) but also with GDS (*n* = 6), DDecPP (*n* = 8), farmers (*n* = 7), and others (*n* = 3). In the majority of cases (91%; *n* = 40), these investigations took <30 min. Three investigations took 1–2 h and one more than 2 h. In four cases, further investigations would have been necessary in light of the severity of the alarm, the excess mortality, and the first telephone investigations. In 33 cases, the results and initial investigations did not reveal whether further investigations would have been useful. The motive to carry out an investigation was essentially based on the criteria of alarm severity (50%), the number of alarm areas (26%), and the distribution of the mortality by age group (17%). The type of alarm (sudden or gradual), proportion of excess mortality, and number of farms with mortality rarely inspired investigation. When no investigation was conducted, the main reasons were the lack of relevance of the excess mortality (78%; *n* = 197), knowledge of the origin of the mortality (welfare or known health problems) (17%; *n* = 43), and also lack of time (12%; *n* = 31). During the field test, despite the incompleteness of data reporting, the system generated many alarms overall, few of which required investigation. These results concord with previous feedback from human SyS systems on the high sensitivity of SyS algorithms (16, 17). Nevertheless, in our case, this sensitivity was acceptable as long as a correct method was implemented for interpreting the results and the organization among animal health services was effective.

Beyond the initial objectives [identification of (re)emergence(s)], our SyS system assists animal health services in their daily missions. It contributed to the monitoring of seasonal diseases, such as influenza or the quantification of the (absence of) impact of bluetongue. It also identified an atypical excess mortality that would otherwise have gone undetected, such as a grouped mortality due to lightning strikes in mountain pastures and the impact of a local salmonellosis problem. In addition, by providing mortality data at the farm level, the system helps health professionals identify or monitor, in real time, farms with welfare or health problems that may require inspection or other support. Finally, feedback indicated that this type of system helps maintain the link between animal health stakeholders, providing an additional opportunity to work together. The lack of reactivity of the system (up to +10 days) was not perceived by users as a limitation. As observed in human SyS systems (12, 18), health professionals are more interested in the follow-up and quantification of the effect of health events rather than early detection.

The main limit identified during the test period was the low amount of reported deaths available at the time of analysis. This low data availability strongly affected the results and the usefulness of the system in two *départements*. Although the high number of alarms in *départements* with the highest data reporting rate first required an adaptation period to find the right analysis method and organization, the main difficulty was handling the frequency of the reports, even though no additional human resources were involved. Another difficulty was the management of alarms in areas overlapping with neighboring non-pilot *départements* when mortality involved livestock outside the *départements*. We encountered this situation especially in *départements* with low cattle density, where many municipalities were merged with municipalities in neighboring *départements* with higher cattle density. Finally, the NC-OMAR could not fully play its role due to insufficient human resources (less than one person for scientific and technical support and coordination). This lack of personnel limited the use of national and departmental results, due to insufficient support for pilot *départements* in interpreting results and reporting. On the other hand, the lack of overall coordination led to a lack of knowledge of the system. Therefore, veterinarians and farmers have little knowledge of the system, which prevents their active participation in the SyS system.

## Perspectives

Despite the limitations and the lack of dedicated resources, nine *départements* decided to continue to assess the effectiveness of the improvements discussed during the mid-term and final meetings. Regarding data reporting, in addition to the discussions between the three national rendering companies and the NC-OMAR, local health services will interact with the collection centers to try to improve data reporting in their *départements*. In addition, data analysis will be delayed 1 week (*w*+2 instead of *w*+1) to work on more complete information, and obtain more realistic results, because responsiveness is not a priority for animal health services at present, due to the current lack of resources needed to react quickly. In the current situation, we hope that in the event of a rapid increase in mortality, local health services will be alerted by farmers, veterinarians, and/or renders, especially as they are used to communicating and working together. This context explains that the current priority is the quality of information to support health services in their daily work and to provide a qualitative quantification of the effect of known events.

To improve the performance of the system, we will add additional seasonality to the GLM, extend the number of weeks guard, and remove the lowest thresholds of the detection algorithms if the increase in data reporting rate leads to a strong increase in alarm areas. To facilitate work in low-bovine-density *départements* (Eure-et-Loire and Yonne), manual spatial aggregation will be set up based on the advice of the local animal health services to test the system under appropriate conditions in these *départements*. Finally, a farm-level analysis will be implemented, to identify farms with abnormally high mortality rates compared to their usual mortality. This monitoring will supplement the current analysis carried out at the area level. It will be helpful to monitor mortality in near-real time in each farm, to better identify farms in difficulty or with health problem when no signal is detected at the area level. It will also help guide investigations to prioritize farms to investigate in alarm areas. This part of the system is eagerly awaited by health professionals and has been delayed due to lack of coordination resources.

## Conclusion

To our knowledge, our bovine SyS system, OMAR, is unique in its organization. It is designed with and for its users in the field and so meets the expectations of stakeholders. This likely explains its success despite the lack of resources and the time that local health services needed to adapt to this new type of surveillance system. Unlike in other SyS systems for which information is available, the interpretation of results is not done at the central level but is based on the expertise on the field (9, 10, 22). This is probably the first time that a SyS has been evaluated in the field in a continuous and systematic way, allowing statistical results to be compared with the actual situation week after week. This feedback, which is an integral part of monitoring systems (23), is rarely carried out because it is time-consuming and organizationally demanding. In our case, feedback revealed unexpected uses of the system by animal health services consistent with their health and surveillance missions. For example, the individual farm mortality data provided in detailed reports are used to monitor the mortality in farms where health control plans have been implemented to assess their effectiveness and ensure that the situation is under control. This demonstrates the usefulness of the system beyond SyS. These unexpected uses are very important to maintain the effectiveness of the system “in peacetime” and the motivation of health services. The 1-year test, in the absence of significant health events, did not provide sufficient precise information on human and financial resources and the main challenge remains to ensure regular working time. It is expected that this evaluation will take several years. Nevertheless, although the demonstrated interest in the system initially compensated for the limited resources, the system is reaching its limits and additional human, financial, and legal resources are now needed to ensure the sustainability of OMAR.

## Data Availability Statement

The data used by the project can be requested from the French Ministry of Agriculture.

## Author Contributions

CS wrote the article, managed, and analyzed the data and co-coordinates the system. J-LV managed the data and automated the analyses. FP and YL are members of the OMAR coordination office and participated in discussions. DC was a member of the OMAR coordination office and coordinated the ESA Platform. CD is a member of the coordination office, coordinates the ESA Platform, and participated in discussions. EG and AT participated in discussions and co-coordinated OMAR.

### Conflict of Interest

The authors declare that the research was conducted in the absence of any commercial or financial relationships that could be construed as a potential conflict of interest.

## Supplementary Material

Supplementary Material S1 Structure of the short online questionnaire for the feedback.

Supplementary Material S2 Rules of the spatial aggregation algorithm.

Supplementary Material S3 The S4 object.

Supplementary Material S4 Detailed report.

Supplementary Material S5 Distribution of the time elapsed between the removal request (death of the animal) and data reporting to the FSDI during the test period for each collection point (based on 1,470,422 removals).

Supplementary Material S6 Weekly distribution of the percentage of the deaths reported in the FSDI at the time of analysis in the surveillance areas in each pilot *département*.
