# Supplementary material for: Designing a Syndromic Bovine Mortality Surveillance System: Lessons Learned From the 1-Year Test of the French OMAR Alert Tool
Source: Front Vet Sci. 2020 Jan 9;6:453. doi: 10.3389/fvets.2019.00453 (PMC6962143; doi:10.3389/fvets.2019.00453)
Supplement: Supplementary Material S1 — Structure of the short online questionnaire for the feedback. [file Table_1.DOCX]

**S1- Structure of the short online questionnaire for the feedback**

Report ID

DD_AAAAAA-WSS

Has the report been consulted?

Yes in full (map and spreadsheet)

Yes, but only the summary report (map)

No

Why was the report not consulted?

*main reason*

No zone in alarm

Due to lack of time

Alarm levels were not relevant

Due to difficulties in accessing reports

Other, specify if you wish

How long did it take to analyze the report?

*Do not count the time spent on investigations, only that dedicated to the study of the report itself.*

Less than 10 minutes

10 to 20 minutes

20 to 30 minutes

30 minutes to 1 hour

1 to 2 hours

More than 2 hours

Have telephone or other investigations been carried out following the analysis of the report?

Yes

No

If not, for what reason(s)

Lack of time

Not necessary in view of the results

Known mortality related to a known farm(s) for an animal protection problem

Known mortality linked to one or more farms under health monitoring

Other, please specify

If yes, with which persons or structures were these telephone or other investigations carried out?

GDS

DDPP

Breeder(s)

Veterinarians(s)

Other, please specify:

How long did these investigations take?

Less than 10 minutes

From 10 to 20 minutes

From 20 to 30 minutes

From 30 minutes to 1 hour

From 1 to 2 hours

More than 2 hours

Would extensive investigations (in the sense of a veterinarian's mandate in the field) have been necessary in certain areas or farms?

Yes

No

We couldn't determine that

On what criteria did you consider that no further investigation was necessary?

*Several possible answers*

Severity of alarm(s)

Alarm type (slow/fast)

Number of zones in alarm

Number of farms with mortality

Excess number of deaths

Proportion of excess mortality

Distribution of mortality by age group

Link to a known animal welfare problem

Following telephone or other investigations

Other, please specify

How many areas would have required a thorough investigation?

For each area indicate the selection criteria:

Alarm severity

Excess mortality

Alarm type (L/R)

Age distribution of mortality

Following telephone or other investigations

No. of farms with mortality

Number of deaths in excess

Other
