# Supplementary material for: Designing a Syndromic Bovine Mortality Surveillance System: Lessons Learned From the 1-Year Test of the French OMAR Alert Tool
Source: Front Vet Sci. 2020 Jan 9;6:453. doi: 10.3389/fvets.2019.00453 (PMC6962143; doi:10.3389/fvets.2019.00453)

**S2 - Rules of the spatial aggregation algorithm**

The algorithm is based on the spatial files provided by the French National Institute of Geographic and Forest Information and NCR data. The main rules are:

- two municipalities merge if they are contiguous (at least two connected x, y points); due to ordination of the municipalities code, municipalities of the same *département* merge preferentially before two of different *départements*,

- two municipalities merge if they both have at least one animal recorded in the NCR database; this limits the integration of urban areas and the grouping of municipalities separated by an urban area,

- grouping is done primarily between two municipalities whose merger makes it possible to obtain a number of animals immediately greater than or equal to the defined threshold (3000 in our case); thus a municipality with 1000 animals contiguous to a municipality with 5000 animals and one with 2500, will merge preferentially with the municipality with 2500 heads. This slows the increase in the number of animals per spatial unit to obtain, insofar as possible, a normal distribution of the cattle population over the new spatial units (surveillance areas) (see Figure below).


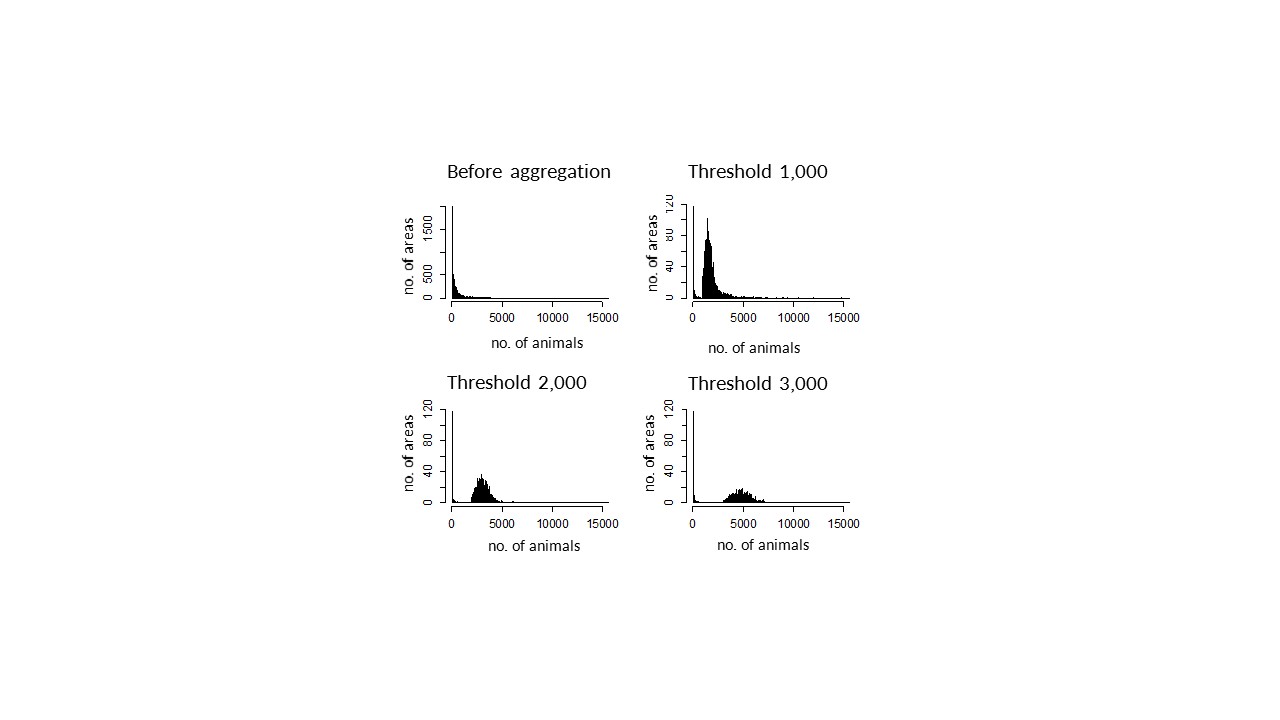

Supplement: Supplementary Material S2 — Rules of the spatial aggregation algorithm. [file Table_2.DOCX]
