# Supplementary material for: Designing a Syndromic Bovine Mortality Surveillance System: Lessons Learned From the 1-Year Test of the French OMAR Alert Tool
Source: Front Vet Sci. 2020 Jan 9;6:453. doi: 10.3389/fvets.2019.00453 (PMC6962143; doi:10.3389/fvets.2019.00453)

### S3 - The S4 object

We adapted the R codes to include automated selection of the cleaning model, the ability to extend the analysis beyond the previous week and the addition of the historical limit algorithm (Figure S3.1).

To initialize the OMAR system, we use the ISO week of the day of analysis and a timetable to record (in the “*time”* slot of the S4 object) the time information needed for the analysis and interpretation of results (ISO week, date of the first day of the week). Then, TS are recorded in the *“observed”* slot. To gain calculation time, only TSs having at least one death recorded in the FSDI over the last four weeks (w_-3_, w_-2_, w_-1_, w) are recorded.

The clean baseline is stored in the *“baseline”* slot, and information on the model (family, formula, over dispersion test result, convergence indication) are recorded in the *“model”* slot.

The results of the detection of excess mortality for each STS and algorithm are recorded in the *“alarm”* slot (one matrix per algorithm results, one row per week w_-3_…w. In addition, the predicted value of the lowest number of deaths exceeded by the observed value is recorded in the *“predicted”* slot for the interpretation of results. For STS with a median number of deaths under one, for consistency of results, for each STS, all the matrices in the *alarm* slot are simultaneously incremented with the same result even if only one method is used.

Figure S3.1: Schematic of the S4 object constructed for data analysis and recording results


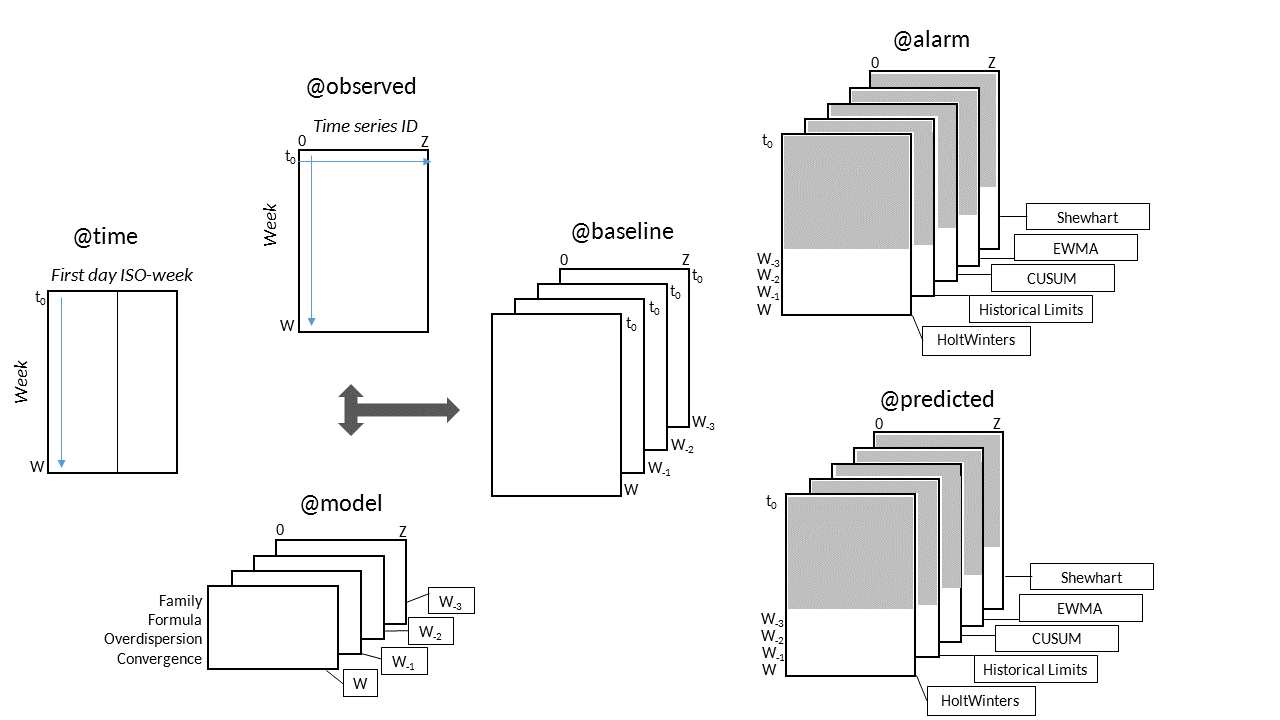

Supplement: Supplementary Material S3 — The S4 object. [file Table_3.DOCX]
