# Supplementary figures and images for: Designing a Syndromic Bovine Mortality Surveillance System: Lessons Learned From the 1-Year Test of the French OMAR Alert Tool
Source: Front Vet Sci. 2020 Jan 9;6:453. doi: 10.3389/fvets.2019.00453 (PMC6962143; doi:10.3389/fvets.2019.00453)

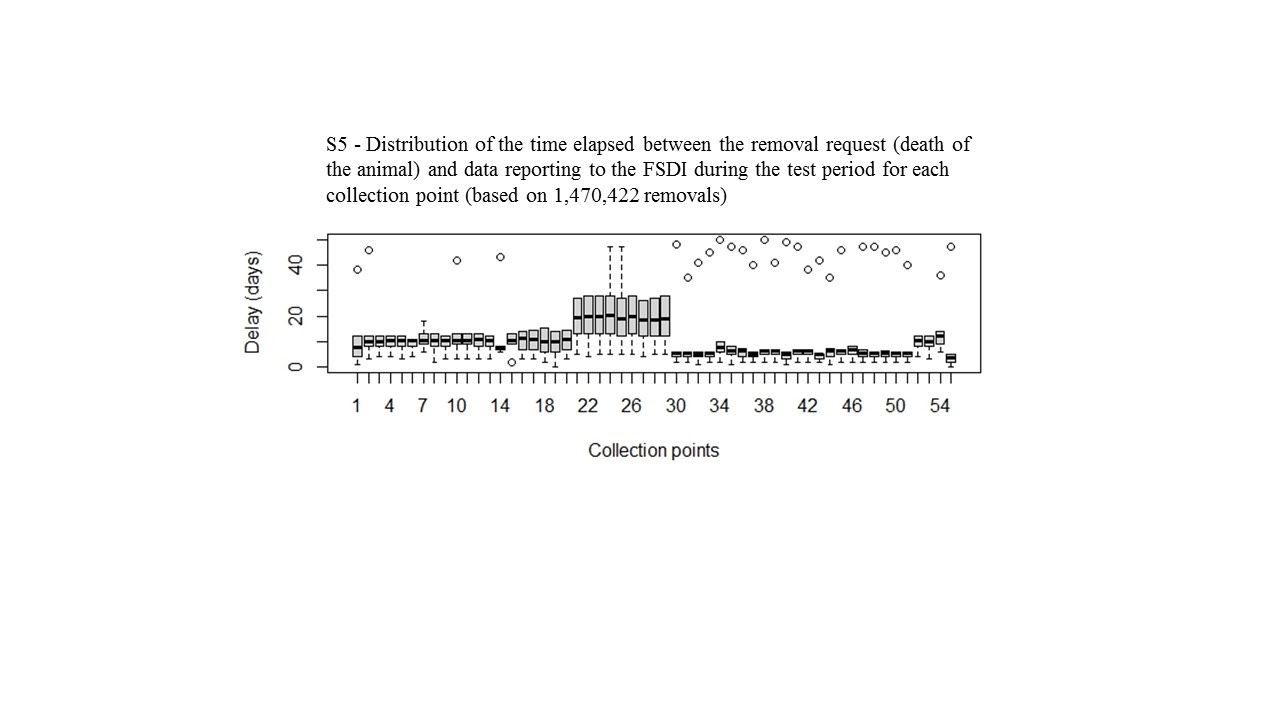

Supplement: Supplementary Material S5 — Distribution of the time elapsed between the removal request (death of the animal) and data reporting to the FSDI during the test period for each collection point (based on 1,470,422 removals). [file Image_1.JPEG]

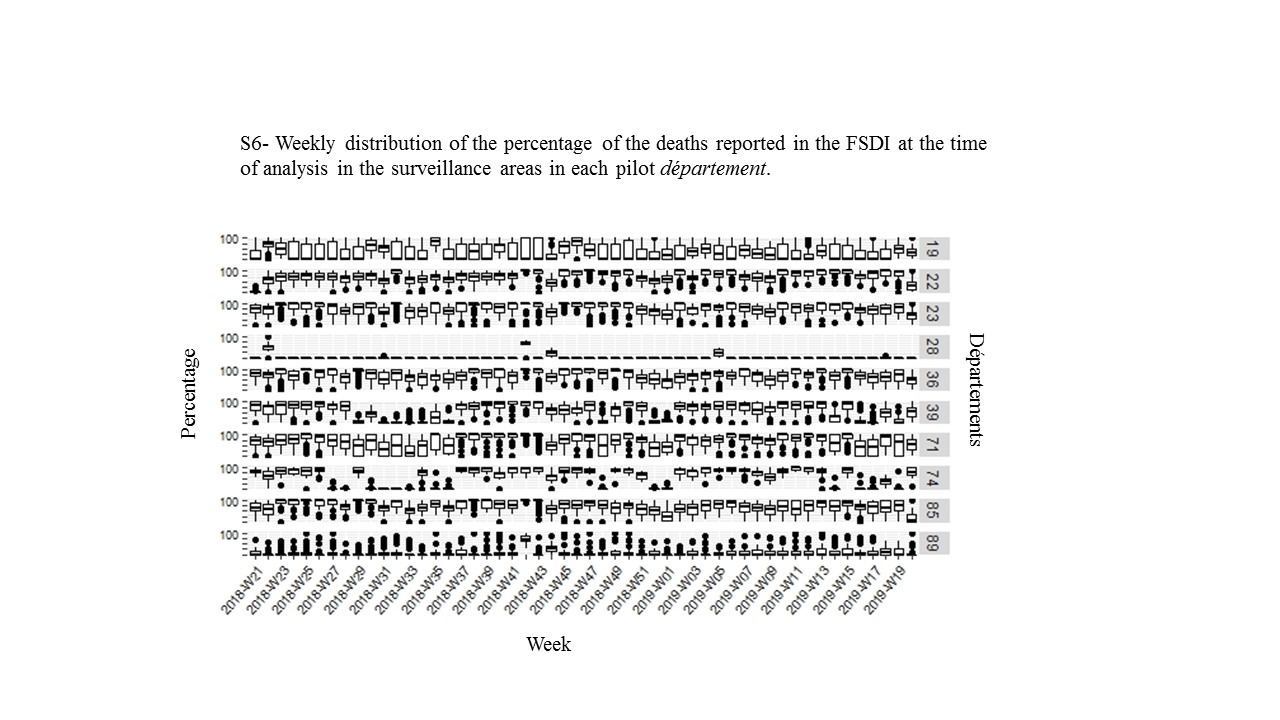

Supplement: Supplementary Material S6 — Weekly distribution of the percentage of the deaths reported in the FSDI at the time of analysis in the surveillance areas in each pilot département. [file Image_2.JPEG]
